# Using ß-cyclodextrin and Arabic Gum as Wall Materials for Encapsulation of Saffron Essential Oil

**Published:** 2017

**Authors:** Mohsen Atefi, Kooshan Nayebzadeh, Abdorreza Mohammadi, Amir Mohammad Mortazavian

**Affiliations:** a ^*a*^*Department of Food Science and Technology, National Nutrition and Food Technology Research Institute, Faculty of Nutrition Sciences and Food Technology, Shahid Beheshti University of Medical Sciences, P.O. Box 19395-4741, Tehran, Iran.*

**Keywords:** Saffron essential oil, Safranal, Encapsulation, ß-cyclodextrin, Arabic gum

## Abstract

Saffron essential oil has a pleasant aroma and medicinal activities. However, it is sensible into the environmental condition. Therefore, it should be protected against unwanted changes during storage or processing. Encapsulation is introduced as a process by which liable materials are protected from unwanted changes. In the present study, different ratios (0:100, 25:75, 50:50, 75:25, and 100:0) of ß-cyclodextrin (ß-CD) and arabic gum (GA) were used as wall martial for encapsulation saffron essential oil. In order to calculate of loading capacity (LC) and encapsulation efficiency (EE), and release (RE), safranal was determined as indicator of saffron essential oil using GC. According to the results, the highest LC and EE were related to the mixture of ß-CD/GA at a 75:25 ratio. In contrast, the lowest encapsulate hygroscopicity (EH) and RE were observed when only ß-CD was applied as wall material (P≤0.05). Comparing the differential scanning calorimetry (DSC) thermograms of the control and encapsulate of ß-CD/GA (75:25) confirmed encapsulation of saffron essential oil. Scanning electron microscopy (SEM) images with high magnifications showed the rhombic structure that partially coated by GA. The mixture of ß-CD/GA at a 75:25 ratio can be recommended for saffron essential oil encapsulation.

## Introduction

Saffron, the dried stigmas of Crocus Sativus Linn. flowers, is the most expensive spice in the world. The plant’s history as a spice began about 2500 years ago, probably in the Mediterranean region and Persia (1, 2). 

The saffron aroma is due to its essential oil, of which safranal is the major volatile compound (approximately 30 to 70 %) (3). Based on different researches, this compound exhibits different pharmacological activities (for example anticonvulsant effect, hypnotic effect, anti-anxiety and anti-stress actions), which justify its importance as a drug of the future (4, 5). Safranal is a monoterpene aldehyde, and the other main aroma compounds also have monoterpenoid structure. 

These compounds are sensitive to light, oxidation and heat (6, 7). Solvent extraction (SE) (with diethyl ether or diethyl ether: pentane), steam distillation (SD), vacuum head space distillation (VHSD), and supercritical fluid extraction (SFE) have been used for extraction of saffron essential oil (8, 9). A Soxhlet extractor can be used to extract of saffron essential oil. This method of extraction is thorough because the cooled organic solvent continuously passes through the target solid sample for a long time. Using the appropriate solvent is critical in this method. Diethyl ether is a suitable candidate solvent for extraction of saffron essential oil, because of its low boiling point (37 °C) and ability to dissolve the saffron essential oil compounds. This offsets the disadvantages of this method, such as poor recovery and destroying of heat-labile compounds (10). 

Saffron essential oil is highly unstable and very prone to absorb oxygen and become thick and brown. Also, it is sensible into the heat and light. Therefore, it is not commercially available and an alcoholic tincture of saffron is used instead for flavor and perfumery› purposes (6, 7). Thus, it should be protected during storage and industrial processing. Encapsulation is a process by which solid particles, liquid droplets or gas cells are covered by a thin layer of wall material (11). Choosing suitable material for encapsulation is important. Flavor or oil encapsulant material should have specific features such as high emulsifying power; good ability to form film; low viscosity at high concentrations; low hygroscopicity and release; low price; desirable taste; sustainability in procurement; and good protective properties (12). No substance has all these features. 

Therefore, to achieve the best possible combination of features, a mixture of wall materials may be an appropriate solution. ß-cyclodextrin (β-CD) is an inexpensive, routine CD consisting of seven glucopyranose units in the form of a truncated cone. The inside of this molecule is hydrophobic, and the outside is hydrophilic. As a result, this structure is more suitable for entrapping hydrophobic compounds. So that, β-CD has been proposed as an appropriate carrier for encapsulation of essential oil compounds (13). Inclusion of monoterpenoids (linalool, S-carvone, camphor, geraniol, *γ*-terpinene fenchone, phenylpropanoids, E-anethole and estragole) with β-CD by precipitation method was done. The results showed that β-CD is a good carrier for monoterpenoids. However, different inclusion efficiencies were observed among the monoterpenoids (14). Arabic gum (GA) is a heteropolysaccharide consisting of high branches linked through 1, 6-galactopyranose residues containing galactose, arabinofuranose, arabinopyranose, rhamnopyranose, glucuronic acid and 4-O-methyl glucuronic acid. This gum has excellent emulsifying properties, film forming ability, good ability of producing small size particle, and low viscosity at high concentrations (15-18). However, GA is not suitable for encapsulation of monoterpenoids when it is used alone (19). Encapsulation of d-Limonen and ethyl n-hexanoate by inclusion method with ß-CD, ß-CD/GA, and ß-cyclodextrin/maltodextrin (ß-CD/MD) was investigated. Using the mixture of ß-CD/GA instead of the ß-CD/MD mixture or ß-CD alone led to higher efficiency (20). 

Saffron essential oil encapsulated with tween 80 and Chitosan has shown a glass-like amorphous structure. Also, the reduction of peak areas from saffron essential oil to encapsulated were 10 % to 58.08 % for eight major compounds (for safranal 10 %) (21).

Up to now, encapsulation of saffron essential oil with different ratios of ß-CD and GA has not been performed. So that, the aim of the present study was to use different ratios of ß-CD and GA to encapsulate saffron essential oil in order to determine the best formulation for retaining of safranal as indicator of saffron essential oil.

## Experimental

*Materials and Methods*

Saffron of first grade was donated by the Iranians Saffron and Cumin Seed Agro-industrial Co., (Mashhad, Iran). Safranal (purity ≤ 88 %) and ß-CD were purchased from Sigma*-*Aldrich Corp. (St. Louis, MO, USA). GA was obtained from Saadatchemieazma Co. (Tehran, Iran). Diethyl ether, ethanol, petroleum ether, and hexane were purchased from Merck Co. (Darmstadt, Germany).

Determination of safranal was performed with a gas chromatography (GC), Agilent Technologies (USA) model 7890A, equipped with a flame ionization detector (FID) and, capillary column HP-5 (30 m × 0.32 mm × 0.2 µm). The temperature was programmed from 60 to 200 °C, increasing at 5 °C min^-1^. The rate of nitrogen flow was set at 15 mL min^-1^. Injection-port and detection temperatures were 250 and 280 °C respectively, with a split ratio of 1:50. The amount of injection volume was 5 μL (with a 10 μL Hamilton syringe).

For extraction of saffron essential oil, 50 g of saffron was ground and extracted in a Soxhlet extractor with 500 mL of diethyl ether for 35 min. The extract was concentrated using rotary evaporator and volume adjusted to 50 mL. This extract was used for determining safranal content, DSC termogram and encapsulation.

In order to prepare of emulsions, different amounts of GA (0, 0.75, 1.5, 2.25, and 3 g) were separately dissolved in 150 mL distilled water and stirred for 30 min with magnetic stirrer at 700 rpm. The solutions were kept overnight at room temperature to the complete hydration of the GA. In contrast, ß-CD solutions were prepared by dissolving different amounts of ß-CD (3, 2.25, 1.5, 0.75, and 0 g) in 250 mL distilled water on the day of encapsulation. For encapsulation, 45 mL of diethyl ether extract was adjusted to 100 mL with ethanol. Then, 20 mL portions of diluted extract were added to each of the ß-CD solutions and stirred for 45 min at 700 rpm with magnetic stirrer. After that, according to the ratios, the solutions of GA were added into the previous solutions and stirred for 45 min. Freezing was done by putting solutions in metal cans that were passed through a freezing tunnel (Aarang Co. Iran) for two hours at -40 °C. They were then dried in the freeze-drier (Cuddon Co. FD 80, Blenheim, New Zealand) for 14 h. The initial temperature of the freeze-drier was -22 °C; during 8h, the temperature was increased to +20 °C. Finally, the temperature was continuously increased to +30 °C after 6 h. The obtained fine powders were kept in sealed containers until further analysis.

To determine of encapsulate hygroscopicity (EH), 0.20 ± 0.0010 g of the encapsulates were spread evenly on Petri dishes (9 cm diameter). They were placed in a desiccator containing saturated NaCl solution (75 % relative humidity) at laboratory temperature (23 ± 2 °C). After 2h, the samples were weighted and hygroscopicity was calculated by difference of initial and final weights (22).

In order to calculate the encapsulation efficiency (EE) and release (RE), the superficial, total and the release safranal amount of encapsulates were determined. For superficial determination of safranal, 0.20 ± 0.0010 g of the encapsulates were weighed and transferred into glass tubes. Then, 20 mL of diethyl ether: petroleum ether (50:50 v/v) was added and mixed by a shaker at 1000 rpm for 1 min. After extraction, solutions were filtered through filter papers (pore size: 34-42 μM). The solvent was then evaporated at 40 ± 2 °C in a warm bath away from light, and the final evaporation (5 mL) was done under nitrogen flow. This solvent-removal procedure was used to determine total and RE of safranal. To determine the total safranal content in the encapsulates, 0.20 ± 0.0010 g of powders were weighed and transferred into screw cap 100 mL dark glass jars. Then, 60 mL of ethanol solution (20 % v/v) was added and stirred using a magnetic stirrer at 1000 rpm for 25 min. After the encapsulates were decomposed, solutions were filtered through cellulosic filter papers and extraction was done on 30 mL of solutions twice with 60 (2×30) mL of diethyl ether: petroleum ether (50:50 v/v) in a separating funnel. To determine released content of safranal, 0.20 ± 0.0010 g of encapsulates were weighed and mixed with 60 mL of water, and the solutions were incubated for 24 h at 23 ± 2 °C in open 100 mL dark glass jars. After that, filtration and extraction were performed to determine the total safranal release. 

The loading capacity (LC %), encapsulation efficiency (EE %) and release (RE %) were calculated according to the researches of Wang and Weller (2006) (Equation 1) with slight modification and Davidov-Pardo D. *et al.* (2013) (Equations 2 and 3) : 

(1) $$\mathrm{LC}(\%)=\left(\frac{\mathrm{The amount of safranal of encapsulate}}{\mathrm{Total safranal in the used extract}\times \frac{1}{5}}\right)\times 100$$

(2) $$\mathrm{EE}(\%)=\left(1-\frac{Su_{E}}{T_{E}}\right)\times 100$$

(3) $$\mathrm{RE}(\%)=\left(\frac{{\mathrm{Rel}}_{E}}{T_{E}}\right)\times 100$$

where

Su_E_ : Superficial content of safranal

T_E_ : Total content of safranal

Rel_E _: Released content of safranal

For differential scanning calorimetry (DSC) a Mettler-Toledo DSC model 822 (Mettler Toledo AG, Switzerland) was employed. 5 mg of saffron essential oil encapsulate with ß-CD/GA (75:25) and its control were weighed with an accuracy of ± 0.01 mg, and placed in a 40 μL closed aluminium pans. In the case of diethyl ether extract, 30 μL was used. Experimental conditions were 10 °C min^-1 ^heating rate from 30 to 300 °C and nitrogen purging at a flow rate of 20 mL min^-1^. An empty pan was used as a reference in all experiments (24).

Scanning electron microscopy (SEM) was used to observe the surface structure. The ß-CD/GA (75:25) encapsulate was mounted on a stub and sputtered with a layer of gold/palladium for 3 min using a sputter coater (Polaron SC7620*,* UK) and examined using a LEO Model 1450 VP (Zeiss, Oberkochen*,* Germany) in a secondary mode at 20 KV accelerating voltages. The quantum was a tungsten-based electron optical column with a resolution of 2 nm. Measurements were taken in vacuum at different magnifications (23).

*Statistical analysis*

Statistical analysis was done using SPSS 18 software (Chicago, IL, USA). The mean values were analyzed by one-way analysis of variance (ANOVA) followed by Duncan’s multiple range tests. A confidence level of 95 % was considered significant. All experiments were performed in triplicate and the results were expressed as mean ± standard deviation (Mean ± SD).

## Results

Calibration curve was linear over the range of 1-500 mg Kg^-1^_. _The precision of the method was evaluated by calculating the relative standard deviation (% RSD) of six replicates by injection of 150 mg Kg^-1 ^safranal solution. The % RSD value was found to be 4.31. The recovery for safranal was determined by comparing the amount of safranal added to an encapsulated sample with the concentration recovered after the procedure. The encapsulated samples spiked with 50 mg Kg^-1^, a line equation of the standard-addition graph, obtained for safranal compound, was applied to the calculation of recovery, which gave 93 %. Also, the limit of quantification (LOQ) and limit of detection (LOD) based on a signal/noise ratio of 3 and 10, respectively were 0.66 and 0.3 mg Kg^-1^, respectively. 

Significant differences were observed in EH values among the formulations. A gradual increase in hygroscopicity was seen when the GA content was increased (P ≤ 0.05) (Table 1.).The results of determining LC varied from 32 to 73 %. Among the formulations, the lowest and highest LC were seen in encapsulates with GA and a mixture of ß-CD/GA (75:25), respectively (P ≤ 0.05) (Table 1.). Also, there were significant differences between the EE of encapsulates. The results of EE ranged from 72 to 98 %. GA and a mixture of ß-CD/GA (75:25) had the lowest and the highest EE, respectively (P ≤ 0.05) (Table 1.). In the RE results, encapsulates using GA and ß-CD had the highest and the lowest RE, respectively (P ≤ 0.05) (Table 1).

**Figure 1:**
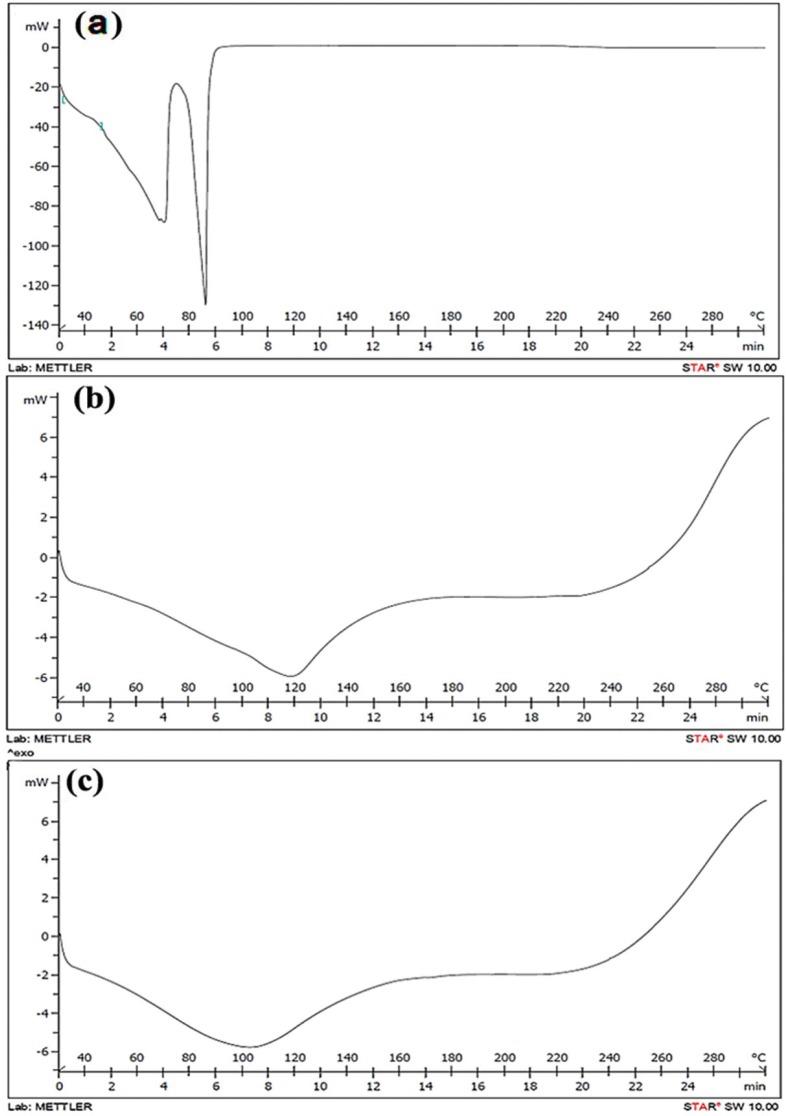
DSC termograms; a: diethyl ether extract, b: control [the mixture of ß-CD/GA (75:25)], c: saffron essential oil encapsulate with ß-CD/GA (75:25

**Figure 2:**
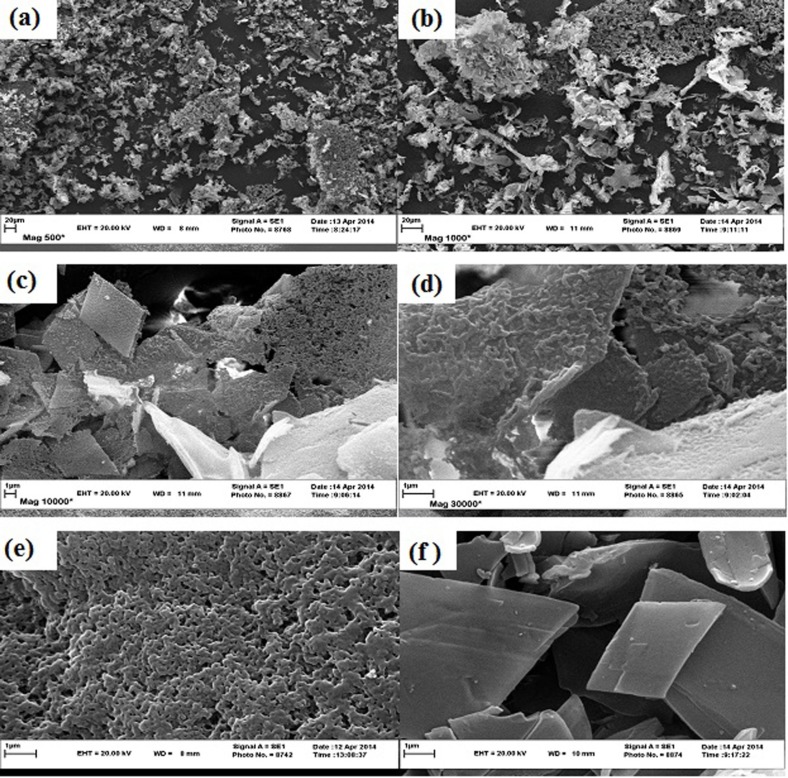
SEM images of saffron essential oil encapsulate with ß-CD/GA (75:25) at different magnitudes (a: magnitude of 500, b: magnitude of 1000, c: magnitude of 10000, and d: magnitude of 30000), and SEM images of GA (e) and ß-CD (f) at magnitude of 30000, respectively

**Table 1:** Determined the mean and standard deviation (Mean ± SD) of encapsulate hygroscopicity (EH %), loading capacity (LC %), encapsulation efficiency (EE %), and release (RE %).

**Sample**	**Encapsulate hygroscopicity (EH %)**	**Loading capacity** **(LC %)**	**Encapsulation efficiency** **(EE %)**	**Release** **(RE %)**
ß-CD/GA (0:100)	10.21 ± 0.65^a^	32.01 ± 3.01^a^	72.49 ± 4.22^a^	66.75 ± 3.23^a^
ß-CD/GA (25:75)	7.89 ± 0.17^b^	43.46 ± 2.53^b^	84.97 ± 4.12^b^	60.61 ± 4.69^a^
ß-CD/GA (50:50)	6.16 ± 0.27^c^	64.29 ± 2.22^c^	89.79 ± 3.26^b^	50.60 ± 1.45^b^
ß-CD/GA (75:25)	4.54 ± 0.45^d^	73.52 ± 3.39^d^	98.39 ± 0.62^c^	41.12 ± 3.99^c^
ß-CD/GA (100:0)	2.39 ± 0.41^e^	60.05 ± 1.85^c^	90.51 ± 1.28^b^	29.12 ± 0.88^d^

a – e Mean ± SD in each column within the same letter are not significantly different

The DSC curve of diethyl ether extract revealed two endothermic peaks corresponding to the melting and vaporization of saffron essential oil compounds (Figure 1a). The first endothermic peak was broad, with the maximum nearly at 70 °C. The second endothermic sharp peak was at 90 °C. 

The DSC curve of the control showed only one broad endothermic peak from 60 to 160 °C, with a maximum at nearly 120 °C (Figure 1b). In comparison to the DSC of the control, the range of the endothermic peak of the ß-CD/GA (75:25) encapsulate remained constant (Figure 1c), while the maximum of the broad peak shifted to 105 °C.

In SEM images of the ß-CD/GA (75:25) encapsulate at magnifications of 500 and 1000, separated porous parts were seen (Figures 2a and 2b). But with increasing the magnification to 10000 and 30000, the rhombic like structure, which covered with a separated porous layer, was revealed (Figures 2c & 2d). In SEM images of GA and ß-CD at a magnitude of 30000, porous layer structure and rhombic shape structure were seen, respectively (Figures 2e and f). 

## Discussion

The encapsulate’s hygroscopicity, or ability to absorb water, is an important physical property. This feature is related to the ability to reconstitute the encapsulates that depends on the molecular interactions between the two phases (25, 26). GA has a hygroscopic nature, because of its high carbohydrate moieties (27, 28). In contrast to GA, ß-CD has a lower hygroscopic nature (29). Therefore, it is reasonable that the EH would increase with the amount of GA in the wall material. Consequently, encapsulations with only GA or ß-CD had the highest and lowest EH, respectively.

The reduction of safranal from the saffron essential oil extract into the encapsulated with tween 80 and chitosan was reported 10 % (9). In our study, in the best formulation, the reduction of safranal was nearly 27 %. The main causes of lower LC in our study, in contrast to previous work, may be due to the kind of wall materials and method of preparation that we used. In preparing ß-CD inclusions, the choice and amount of solvents are crucial. Ethanol and diethyl ether are appropriate solvents for dissolving lipophilic compounds, and water is an essential solvent for dissolving of ß-CD, but optimization is needed for achieving a better outcome (30). 

EE is a more important parameter even than LC, because of the need to retain a high value of essential oil in the encapsulation process (31). The finding of the appropriate ratio of wall materials is necessary for minimizing the loss of the essential oil. ß-CD is used as encapsulate material for the inclusion of monotrepenoids. The results indicated that ß-CD is an efficient wall material for inclusion of monotrepenoids, with EE values ranging from 31 to 51 % depending on the compounds (14). This range of EE values may be related to precipitation and drying with heat they used for inclusion of monoterpens. In an aqueous solution, the ß-CD cavity is slightly polar and occupied by water molecules, and can therefore be readily replaced by appropriate guest molecules, that are less polar than water (24). Most monoterpens have low water solubility; this has been shown to be true for safranal (32, 33). Therefore, either safranal or the other main monotrepenoids of saffron essential oil compounds can act as suitable guest molecules for replacing water molecules in the ß-CD cavity. In contrast to ß-CD, using GA for encapsulation of monoterpens has revealed that it is not efficient as a wall material, because of oxidative processes in the core materials during storage (18). However, GA has been found to be more efficient than sucrose and gelatin for encapsulation of limonene in freeze-drying due to its film-forming ability (16). A combination of ß-CD and GA were shown to offer more protection than ß-CD alone (20). It can be concluded that ß-CD has a higher capacity for encapsulation of monoterpens than GA, and also that a mixture of the two increases encapsulation efficiency. Therefore, the inclusion ability of ß-CD and the film-forming ability of GA is a highly efficient combination for encapsulating monoterpens and similar structures such as safranal. The core material release of the encapsulate takes place by several mechanisms, including surface erosion, disintegration, diffusion and desorption. The main release mechanism of flavor components is diffusion, and it depends on the solubility and permeability of the wall material (34). Water uptake is an important property of encapsulate release, because it changes the matrix structure (35). Greater solubility of wall materials in water may lead to increased release. GA is a complex acidic heteropolysaccharide composed of a highly branched structure of galactose, arabinose, rhamnose, and glucuronic acid; it is highly water-soluble (36). Therefore, it is reasonable that with increasing GA in the formulations, the amount of release increases. In contrast to GA, ß-CD has a low water solubility (1.85 % w/v), and the release of its guest molecule depends on the chemical equilibrium of the complex (13). The RE of our study confirmed this phenomenon: encapsulates using GA and ß-CD had the highest and the lowest RE, respectively. 

DSC is a common method for assessing the formation of a complex in the solid state (37). ß-CD, like other CDs, has no well-defined melting point (13). A broad endothermic peak from 60 to 100 °C (or 120 °C), related to the release of water molecules, has been reported for ß-CD (38, 39). However, in other research, ß-CD has shown a broad endothermic event with a baseline decreasing from close to 90 °C, and with a peak above 170 °C due to water evaporation (24, 37, 40). Also, GA with a low water content (0-40 %) has an endothermic peak at 90 °C due to the release of its water molecules (41). It can be concluded that the new organization system formed from ß-CD and GA increases the melting point in comparison with each alone. Compared to this curve, after encapsulation of saffron essential oil, change in peak position was observed. Change in peak position is an evidence of interactions between the wall materials and saffron essential oil compounds that resulted in the formation of the new structural organization. This shift to a lower temperature may be due to the decreasing effect of saffron essential oil compounds on the melting point of the mixture. In addition, the disappearance of the endothermic DSC signal responsible for the saffron essential oil can be a strong proof of accomplishing encapsulation (37). Nevertheless, the DSC of ß-CD/GA (75:25) encapsulate showed that it hasn’t high heat-stability, and that the gradual release of saffron essential oil compounds occurs along with that of the water molecules in the range of 40 to 120 °C. Most complexes of ß-CD are heat-sensitive, and their decomposition starts at 50 to 60 °C (30). The morphological characteristics of the encapsulate depend heavily on the materials, and on the methods used for drying (16). The freeze-drying method results in, the morphological structure having a continuing porous-layer structure (42). A porous-layer structure was seen in our SEM images of ß-CD/GA (75:25) encapsulate at low magnifications. At higher magnitudes, aggregated parts with a rhombic shape structure that partially coated with a porous-layer structure were seen. Also, in SEM images of GA and ß-CD encapsulates, porous-layer structure and separated rhombic shape parts were seen, respectively. The aggregates with a crystalline structure for ß-CD has been reported by other researchers in aqueous solutions (43, 44). So, it can be concluded that the parts with rhombic shape and porous-layer structure on them are due to aggregation ability of ß-CD and film forming ability of GA, respectively. The SEM images with high magnification clearly indicated that the order in which ß-CD and GA were used affected the morphological structure of the encapsulate, as we used ß-CD before GA. Thus, separated aggregate forms of ß-CD were produced and GA acted as a cover on their surface. It›s worthwhile to mention that for better coverage of ß-CD aggregates containing saffron essential oil, the preparation method for encapsulation should be improved.

## Conclusion

Our findings indicated that increasing EH is in accordance with increases of GA in the mixture of GA/ß-CD encapsulates. Also, a mixture of GA and ß-CD leads to more LC and EE, such that the highest EE was obtained from ß-CD/GA (75:25). According to the RE result, GA increased RE. Therefore, the highest and the lowest releases were related to GA and ß-CD only, respectively. The encapsulation of saffron essential oil led to a shift in the maximum peak of the DSC curve to a lower temperature compared to the control, because of interactions between the wall materials and saffron essential oil compounds. The SEM images at low magnifications revealed separated porous parts, but at high magnifications showed rhombic shape structure in these porous parts. This structure was due to aggregation ability of ß-CD and film forming ability of GA, respectively. 
